# The glucose transporter GLUT1 is required for ErbB2-induced mammary tumorigenesis

**DOI:** 10.1186/s13058-016-0795-0

**Published:** 2016-12-20

**Authors:** Elizabeth A. Wellberg, Stevi Johnson, Jessica Finlay-Schultz, Andrew S. Lewis, Kristina L. Terrell, Carol A. Sartorius, E. Dale Abel, William J. Muller, Steven M. Anderson

**Affiliations:** 1 Department of Pathology, MS 8401, University of Colorado Anschutz Medical Campus, 12801 East 17th Avenue, Box 8104, Aurora, CO 80045 USA; 2 Department of Medicine, Carver College of Medicine, University of Iowa, Iowa City, IA 52242 USA; 3 Department of Biochemistry, McGill University, Montreal, Quebec H3A 1A3 Canada; 4 Rosalind and Morris Goodman Cancer Center, McGill University, Montreal, Quebec H3A 1A3 Canada; 5 Program in Cancer Biology, MS 8401, University of Colorado Anschutz Medical Campus, Aurora, CO 80045 USA; 6 Program in Molecular Biology, MS 8401, University of Colorado Anschutz Medical Campus, Aurora, CO 80045 USA

**Keywords:** Glucose, GLUT1, Slc2a1, MMTV-NIC, Breast cancer, Mammary tumor, HER2, ERBB2, Neu

## Abstract

**Background:**

Altered tumor cell metabolism is an emerging hallmark of cancer; however, the precise role for glucose in tumor initiation is not known. GLUT1 (*SLC2A1*) is expressed in breast cancer cells and is likely responsible for avid glucose uptake observed in established tumors. We have shown that GLUT1 was necessary for xenograft tumor formation from primary mammary cells transformed with the polyomavirus middle-T antigen but that it was not necessary for growth after tumors had formed in vivo*,* suggesting a differential requirement for glucose depending on the stage of tumorigenesis.

**Methods:**

To determine whether GLUT1 is required early during mammary tumorigenesis, we crossed MMTV-NIC mice, which express activated HER2/NEU/ERBB2 and Cre recombinase, to *Slc2a1* ^Flox/Flox^ (GLUT1^Flox/Flox^) mice to generate NIC-GLUT1^+/+^, NIC-GLUT1^Flox/+^, and NIC-GLUT1^Flox/Flox^ mice. In addition, we evaluated effects of glucose restriction or GLUT1 inhibition on transformation in MCF10A-ERBB2 breast epithelial cells in three-dimensional culture. Finally, we utilized global gene expression profiling data of primary human breast tumors to determine the relationship between *SLC2A1* and stage of tumorigenesis.

**Results:**

All of the NIC-GLUT1^+/+^ mice developed tumors in less than 200 days. In contrast, only 1 NIC-GLUT1^Flox/Flox^ mouse and 1 NIC-GLUT1^Flox/+^ mouse developed mammary tumors, even after 18 months. Mammary gland development was not disrupted in NIC mice lacking GLUT1; however, epithelial content of mature glands was reduced compared to NIC-GLUT1^Flox/+^ mice. In MCF10A-ERBB2 cells, glucose restriction or GLUT1 inhibition blocked transformation induced by activated ERBB2 through reduced cell proliferation. In human breast cancers, *SLC2A1* was higher in ductal carcinoma in situ compared to the normal breast, but lower in invasive versus in situ lesions, suggesting the requirement for GLUT1 decreases as tumors progress.

**Conclusions:**

This study demonstrates a strict requirement for GLUT1 in the early stages of mammary tumorigenesis in vitro and in vivo. While metabolic adaptation has emerged as a hallmark of cancer, our data indicate that early tumor cells rely heavily on glucose and highlight the potential for glucose restriction as a breast cancer preventive strategy.

## Background

It is well established that cancer cells require abundant glucose, not only to meet the energy demands of rapidly proliferating cells, but also to provide a carbon source for building blocks that generate nucleotides, proteins, cell membrane lipids, and reducing power in the form of NADPH. Glucose utilization by cancer cells has been studied for decades since Warburg’s initial observations in 1924 that tumors perform glycolysis even in the presence of abundant oxygen [1, 2]. Initially, it was thought that cancer cell mitochondria were dysfunctional and that the cells relied on glucose to provide ATP through glycolysis. Now we know that cancer cell mitochondria are capable of providing sufficient ATP through oxidative phosphorylation, and it has been suggested that the majority of glucose carbons are shunted from glycolysis to adjacent pathways for the purpose of generating amino acids, nucleotides, and fatty acids [3]. The heightened cancer cell demand for glucose has been exploited in diagnostic procedures, which rely on positron emission tomography (PET) imaging of tumor tissues that have taken up ^18^F-fluoro-2-deoxyglucose (FDG) [4, 5]. PET scanning has been clinically useful for detecting recurring lesions as well as emergence of metastatic lesions, and increased FDG uptake has been associated with higher grade [6, 7], poorly differentiated tumors [8, 9], and high proliferation rates [8, 10] in breast cancer.

Glucose enters cells through various transporters including the GLUT family of facilitative transporters, the sodium/glucose co-transporters, and the recently discovered SWEET family [11]; however, only the role of the GLUT family has been extensively studied in cancer cells. There are 14 GLUT proteins in the human and 12 in the mouse [12]. GLUT1, encoded by *SLC2A1*, is the predominant transporter overexpressed in tumors, including hepatic, pancreatic, esophageal, brain, renal, lung, cutaneous, colorectal, endometrial, ovarian, cervical, and breast, as well as head and neck tumors (reviewed in [13]). In addition to GLUT1, expression of GLUTs 2–5 has been observed in breast tumors, but levels of these transporters are variable (reviewed in [13]). In breast cancer, the extent of tumors positive for GLUT1 by immunohistochemistry varies from 42% to greater than 90% [7, 10, 14–16]. Although it is not clear when GLUT1 expression is increased during tumorigenesis, Alo et al. observed that 36% of typical/atypical hyperplastic breast tissue expressed GLUT1 and normal adjacent tissues were positive in 31% of cases, suggesting that overexpression of GLUT1 may occur early in the transformation process [14]. Pinheiro et al. evaluated GLUT1 levels in 124 cases of breast cancer that were stratified by subtype, receptor status, and other molecular markers, and demonstrated that GLUT1 was associated with increased tumor size, higher tumor grade, higher rates of proliferation, and was predominately expressed in the basal subtype [17]. GLUT1 expression was also associated with increased expression of carbonic anhydrase, perhaps reflecting the altered metabolism in these tumors [17]. It is unclear whether GLUT1 levels correlate with FDG uptake, although PET scanning remains clinically important in the management of breast cancer [8, 18].

GLUT2 and GLUT3 [19] have been detected in approximately 30% of invasive breast cancer tissues, and GLUT4 appears to be expressed in even fewer cases [19, 20]. GLUT12 was cloned from MCF7 cells [21] and was found to be expressed at relatively higher levels in mammary tumors compared to surrounding normal epithelial cells [22]. There are conflicting reports of GLUT5 levels in breast cancer, with studies showing that GLUT5 is [23] and is not [24] overexpressed in primary breast cancer samples and cell lines.

Expression of GLUT1 is induced by hypoxia [25, 26], c-myc [27], and by Akt1 [28]. A complex of hypoxia inducible factor 1α (HIF1α) and aryl-hydrocarbon receptor nuclear translocator (ARNT) has been shown to bind to the GLUT1 promoter and upregulate its expression [25]. In addition, estrogen, acting through the estrogen receptor, has also been shown to induce GLUT1 levels in breast cancer cells [29]. The majority of triple-negative breast cancers have high levels of GLUT1 as measured in tissue microarrays [30].

We previously showed that GLUT1 was the most abundantly expressed glucose transporter in cell lines derived from ERBB2/NEU-induced mouse mammary tumors and that shRNA-mediated reduction of GLUT1 in mouse mammary tumor cell lines decreased glucose uptake and proliferation without altering ATP levels [31]. When these cells were grown as xenograft tumors, the growth rate of GLUT1 knockdown cells was approximately half of the controls, suggesting that GLUT1 and potentially glucose uptake were not critical to support the growth of tumors once they were established. These studies were extended using mammary epithelial cells isolated from mice bearing floxed alleles of *Slc2a1* (referred to as GLUT1^F/F^) and transformed with polyomavirus middle-T antigen (PyMT) in vitro. Cre-mediated *Slc2a1* excision completely prevented tumor formation when cells were grown as orthotopic xenografts. When *Slc2a1* was excised after the cells had grown as tumors in vivo (i.e., when cells were cultured from control tumors and then treated with Cre recombinase), a decrease in tumor growth rates and glucose uptake was observed in the absence of GLUT1, but tumors formed. These studies suggested that GLUT1 expression and glucose uptake are early, obligate events for mammary tumor formation.

In the current study, we crossed the MMTV-NIC (mouse mammary tumor virus promoter-Neu-IRES-Cre) transgenic mouse line to GLUT1^F/F^ mice [31]. Here, we show that loss of one or both alleles of *Slc2a1* from mammary epithelial cells expressing the active Neu oncogene prevented tumor formation. In addition, blocking GLUT1 or restricting available glucose led to decreased cell proliferation and suppressed features of transformation in MCF10A cells expressing a conditionally active human epidermal growth factor receptor 2 (HER2/NEU/ERBB2) construct (MCF10A-ERBB2). These studies confirm that restricting glucose uptake inhibits mammary tumorigenesis in ERBB2-induced models and support the development of preventive strategies for breast cancer based on targeting glucose metabolism.

## Methods

### Mice

Transgenic MMTV-NIC (Neu-IRES-Cre) mice were kindly provided by Dr. William Muller (McGill University, Montreal, Canada) and have been described previously [32]. GLUT1^Flox/Flox^ (GLUT1^F/F^) mice were generated by E. Dale Abel (University of Iowa, Iowa City, IA, USA) as described previously [31], and were backcrossed to the FVB genetic background using a speed congenic approach. All mice used in this study contained greater than 90% FVB alleles as determined by marker analysis (data not shown). Mice were housed in the Center for Comparative Medicine, with ad libitum access to food and water on a standard 12-h light/dark cycle. MMTV-NIC males were bred to GLUT1^F/F^ females, and then NIC-GLUT^F/+^ males were bred to GLUT1^F/+^ females to generate NIC-GLUT1^+/+^, NIC-GLUT1^F/+^, and NIC-GLUT1^F/F^ progeny. Female mice were palpated weekly for mammary tumors beginning at 8 weeks of age. The age of the mouse upon first palpable mammary tumor was recorded for Kaplan-Meier analysis. Tumor studies were carried out to 18 months. All animal studies were conducted in accordance with protocols approved by the Institutional Animal Use and Care Committee of the University of Colorado, Denver, USA.

### Mammary whole mounts

Mammary whole mounts were performed as previously described using standard carmine-alum staining protocols [33].

### Antibodies and immunohistochemistry

Tissues were fixed in 10% neutral-buffered formalin, and processed and embedded according to standard histologic protocols. Five-micron sections were stained with hematoxylin and eosin (H&E) or used for immunohistochemical analysis. The GLUT1 antibody has been described previously [31]. Anti-Cre recombinase antibody was from Thermo Scientific, anti-Ki67 antibody was obtained from Dako, and anti-BrdU antibody was from Abcam. Cre recombinase and Ki67 were quantified by counting the number of positive cells out of the total number of mammary epithelial cells in one full, representative mammary gland section per mouse. BrdU staining was quantified by counting the number of positive cells out of the total cells per acinar structure in 3–4 paraffin-embedded sections per treatment, cut every 50 μm. GLUT1 and tumor area were quantified using the Aperio Digital Pathology system (Leica Biosystems).

### Cell culture

MCF10A-ERBB2 cells were provided by Dr. Senthil Muthuswamy (Harvard Medical School) and cultured as described [34]. Cells were authenticated by short tandem repeat (STR) analysis within 6 months of performing experiments. Cells were mycoplasma negative as determined by the MycoAlert Mycoplasma Detection Kit (Lonza). Matrigel was obtained from BD Biosciences and the ERBB2 construct was activated by addition of the BB homodimerizer (BD Biosciences) at a concentration of 500 nM. WZB117 was purchased from Sigma-Aldrich. The size of MCF10A-ERBB2 acinar structures under different culture conditions was determined at indicated times by capturing digital images of the colonies using a 4× objective on a Nikon Eclipse T*i* microscope. Volumes were calculated from measured diameters and NIS Elements AR software. Matrigel-embedded acinar structures were incubated with 0.25 mg/mL BrdU (Sigma Aldrich) for 1 h, fixed in 4% paraformaldehyde, embedded in Histogel (Thermo Scientific), and embedded in paraffin for immunohistochemical analysis.

### Flow cytometry

All females were in estrus at sacrifice, as determined by examination of cells from vaginal lavage. Whole inguinal (#4) mammary glands were dissected and digested at 37 °C for 4 h in 1 mg/mL collagenase B (Roche) and 0.5 mg/mL hyaluronidase (Sigma Aldrich) with gentle shaking. Dissociated cells were washed and filtered through a 50-μm cell strainer before incubating with antibodies. All antibodies for flow cytometry were purchased from BioLegend. Antibodies to CD31, CD45, and TER119 were conjugated to Pacific Blue. Additional antibodies used were Alexa-fluor 488 anti-CD24, APC anti-CD29, and PE anti-CD61. Samples were analyzed on a Galios (Beckman Coulter). Analysis was performed with Kaluza Analysis Software (Beckman Coulter).

### RNA isolation and quantitative PCR analysis

RNA was isolated from acinar structures using Qiazol (Qiagen). One microgram of total RNA was reverse transcribed using the Verso cDNA synthesis kit (Thermo Scientific). Quantitative polymerase chain reaction (PCR) was performed as previously described [35], using Taqman primers and probe to *SLC2A1* (Life Technologies).

### Human data analysis

The gene expression data from normal breast, ductal carcinoma in situ (DCIS), and invasive ductal carcinoma (IDC) samples are publicly available in dataset GSE59246. Raw data and annotation files from this dataset were downloaded and samples were sorted by tissue type. Log_2_ transformed expression of *SLC2A1* was plotted based on tissue type and analyzed using unpaired *t* tests to compare DCIS to normal breast and IDC to DCIS.

### Statistical analyses

Tumor latencies are shown as Kaplan-Meier survival curves. Because only one animal in the NIC-GLUT1^F/+^ and NIC-GLUT1^F/F^ groups developed a tumor, we were unable to define a median survival time for these groups and we were insufficiently powered to perform robust statistical analyses. For all analyses, we used D’Agostino and Pearson omnibus normality test, and then unpaired *t* tests or non-parametric tests depending on whether data were normally distributed or not. When variances were found to be unequal, this was accounted for in the *t* test using a Welch’s correction. If sample sizes were too small to assess normality, we performed non-parametric tests. The level of significance for all tests was set at 0.05.

## Results

### GLUT1 loss prevents mammary tumor formation without disrupting normal cell growth

**Fig. 1:**
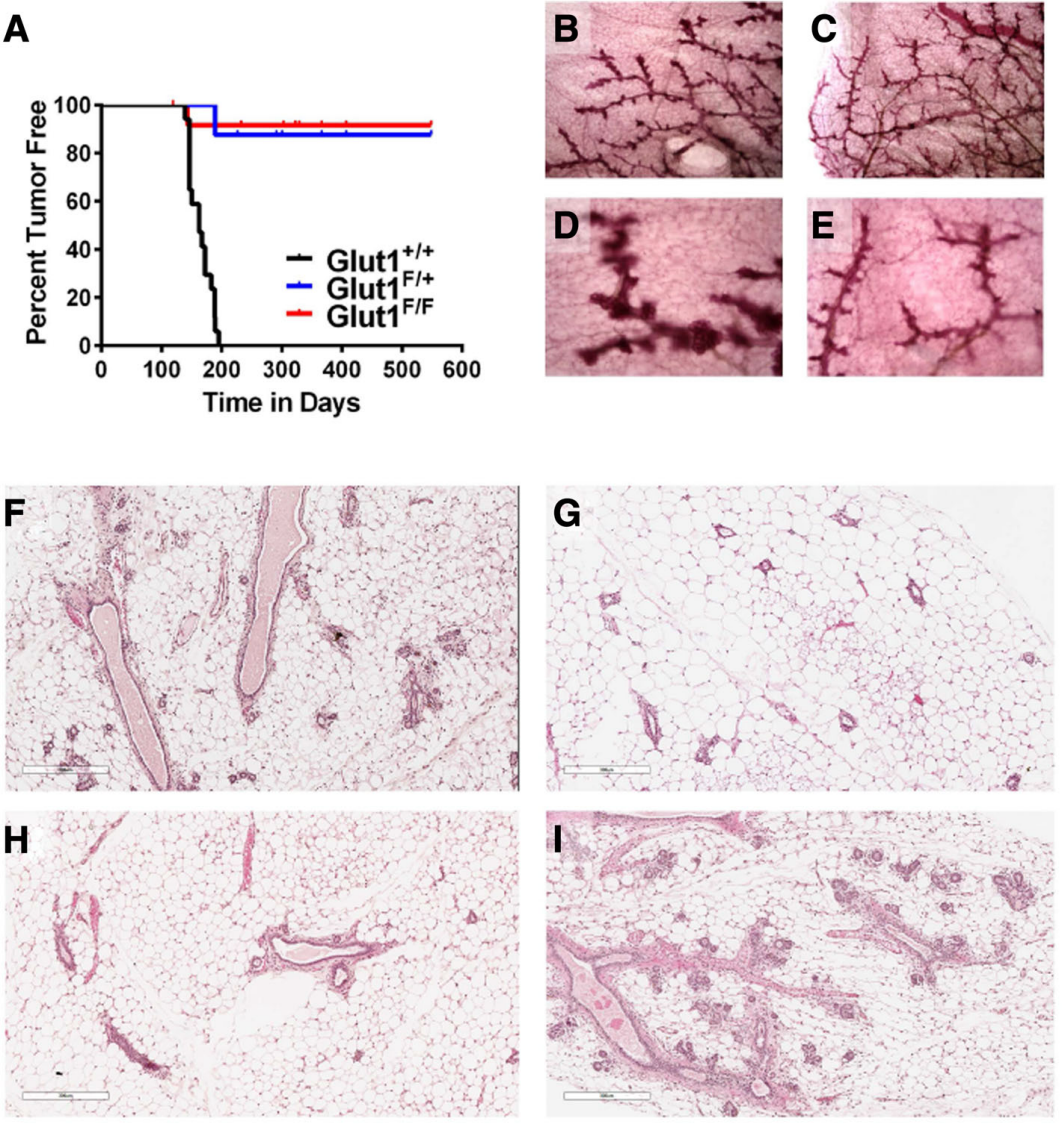
Loss of GLUT1 prevents HER2/ERBB2/Neu-induced mammary tumor development. **a** Kaplan-Meier curve of tumor-free survival in MMTV-NIC mice with wild-type GLUT1 (*Glut1* ^*+/+*^), one floxed GLUT1 allele (*Glut1* ^*F/+*^) or two floxed GLUT1 alleles (*Glut1* ^*F/F*^). *N* = 17, 8, and 13 mice per group, respectively, for GLUT1^+/+^, GLUT1^F/+^, and GLUT1^F/F^. Vertical marks on the curves for NIC-GLUT^F/F^ and NIC-GLUT^F/+^ indicate times at which animals were sacrificed for analysis (censored subjects). Whole mounted mammary glands (**b**–**e**) from NIC-GLUT1^F/+^ (**b**, **d**) and NIC-GLUT1^F/F^ (**c**, **e**) nulliparous adult female mice. Images **b** and **c** were captured at 4× magnification, and images **d** and **e** were captured at 10× magnification. H&E-stained sections of mammary glands from NIC-GLUT1^F/+^ (**f**), NIC-GLUT1^F/F^ (**g**), wild-type FVB (**h**), and NIC-GLUT1^+/+^ (**i**) nulliparous adult female mice. *Scale bars* for H&E images are 300 μm

We previously showed that eliminating GLUT1 from PyMT-transformed primary mouse mammary epithelial cells prior to orthotopic injection prevented tumor formation when cells were implanted into immune-compromised mice [31]. In contrast, when GLUT1 was deleted from cells that had previously grown as xenograft tumors in vivo, glucose uptake and lactate production were reduced; however, these cells lacking GLUT1 formed slow-growing tumors [31]. Based on these studies, we hypothesized that glucose uptake, mediated by GLUT1, is critical for the initial steps of mammary tumorigenesis and the emergence of a mammary tumor. To test this hypothesis, we crossed MMTV-NIC mice to GLUT1^Flox/Flox^ (GLUT1^F/F^) mice, and monitored mammary tumor formation in NIC-GLUT1^+/+^, NIC-GLUT1^F/+^, and NIC-GLUT1^F/F^ progeny. NIC-GLUT1^+/+^ mice developed mammary tumors with a median latency of 162 days and all mice formed tumors by 200 days (Fig. 1a), consistent with previous reports from this model [32]. In contrast, tumor development was delayed in NIC-GLUT1^F/+^ and NIC-GLUT1^F/F^ mice. Only one NIC-GLUT1^F/+^ and one NIC-GLUT1^F/F^ mouse developed tumors, with latencies of 189 and 143 days, respectively (Fig. 1a).

To determine whether GLUT1 excision interfered with the development of the mammary ductal tree, we evaluated epithelial structures in whole mounted mammary glands and H&E sections from mature (4–6 months of age), nulliparous female mice. In both NIC-GLUT1^F/+^ and NIC-GLUT1^F/F^ mice, we observed fully developed mammary glands. NIC-GLUT1^F/+^ mice appeared to have increased epithelial content that appeared like alveolar buds along ducts, which could also represent hyperplasia, but no tumors were observed (Fig. 1b and d). In NIC-GLUT1^F/F^ mice, we observed lower epithelial content compared to NIC-GLUT^F/+^, and no tumors (Fig. 1c and e). In mammary gland sections, we found that NIC-GLUT^F/+^ mice had areas of dense epithelium (Fig. 1f), consistent with what we observed in whole mounts (Fig. 1b and d), but this was not seen in NIC-GLUT1^F/F^ glands (Fig. 1g). In fact, NIC-GLUT1^F/F^ glands more closely resembled those of wild-type FVB mice (Fig. 1h) than those of NIC-GLUT1^F/+^ (Fig. 1f) or NIC-GLUT1^+/+^ (Fig. 1i) mice. Thus, Cre-mediated excision of Glut1 in the adult mouse mammary gland does not appear to disrupt ductal elongation or branching.

**Fig. 2:**
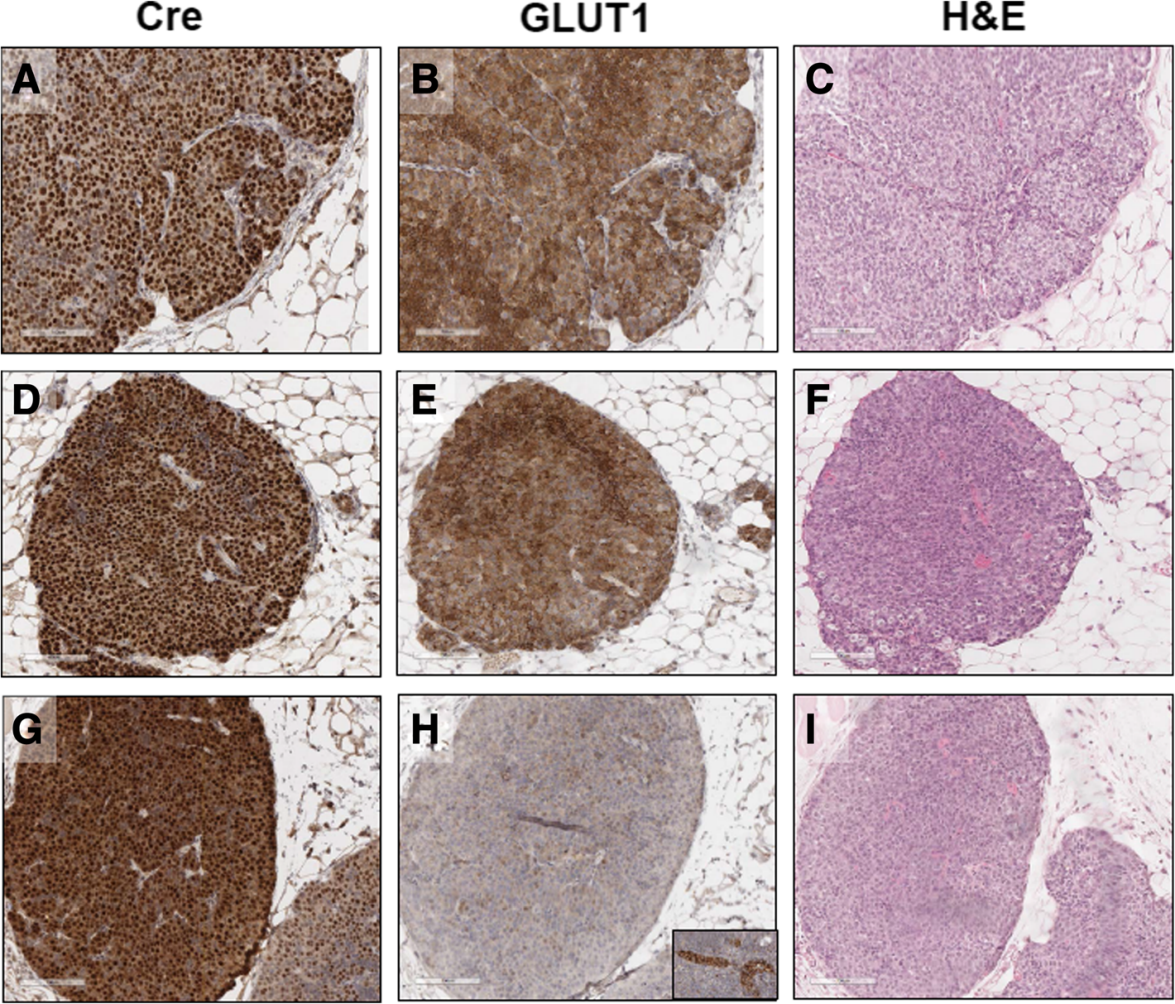
Cre and GLUT1 expression in tumors from NIC mice with and without GLUT1. Representative images of a tumor from a NIC-GLUT1^+/+^ mouse showing Cre staining (**a**), GLUT1 staining (**b**), and hematoxylin and eosin (*H&E*) staining (**c**). Representative image of a tumor from a NIC-GLUT1^F/+^ mouse showing Cre staining (**d**), GLUT1 staining (**e**), and H&E staining (**f**). Representative image of a tumor from a NIC-GLUT1^F/F^ mouse showing Cre staining (**g**), GLUT1 staining (**h**), and H&E staining (**i**). Inset in **h** shows GLUT1-positive cells within the lymph node from the same section containing the GLUT1-negative tumor

The NIC transgene expresses both Neu and Cre recombinase; therefore, we used Cre expression as an indicator of transgene expression. Strong, nuclear Cre expression was detected in all tumors examined from the NIC-GLUT1^+/+^ mice (Fig. 2a), and these tumors also expressed GLUT1 (Fig. 2b). Both Cre recombinase and GLUT1 were detected in tumors from the NIC-GLUT1^F/+^ mouse (Fig. 2d and e). In the tumors from the NIC-GLUT1^F/F^ mouse, we detected Cre recombinase, but not epithelial expression of GLUT1 (Fig. 2g and h). GLUT1 was detected in immune cells within the mammary lymph node from the NIC-GLUT1^F/F^ mouse (Fig. 2h, inset). Histologically, all tumors arising in the NIC mice were consistent with those previously described in this model and in the MMTV-Neu mammary tumor model (Fig. 2c, f, and i) [32]. The resolution of immunostaining is not sufficient to determine the extent of GLUT1 staining in the plasma membrane versus the cytoplasm of tumor cells, although it does appear that some GLUT1 may be cytosolic. Together, these data suggest that GLUT1 is required for the early stages of Neu-induced mammary tumorigenesis, and that loss of only a single copy of *Slc2a1* is sufficient to prevent the majority of Neu-induced mammary tumors.

### Reduced mammary epithelial cell proliferation in the absence of GLUT1

**Fig. 3:**
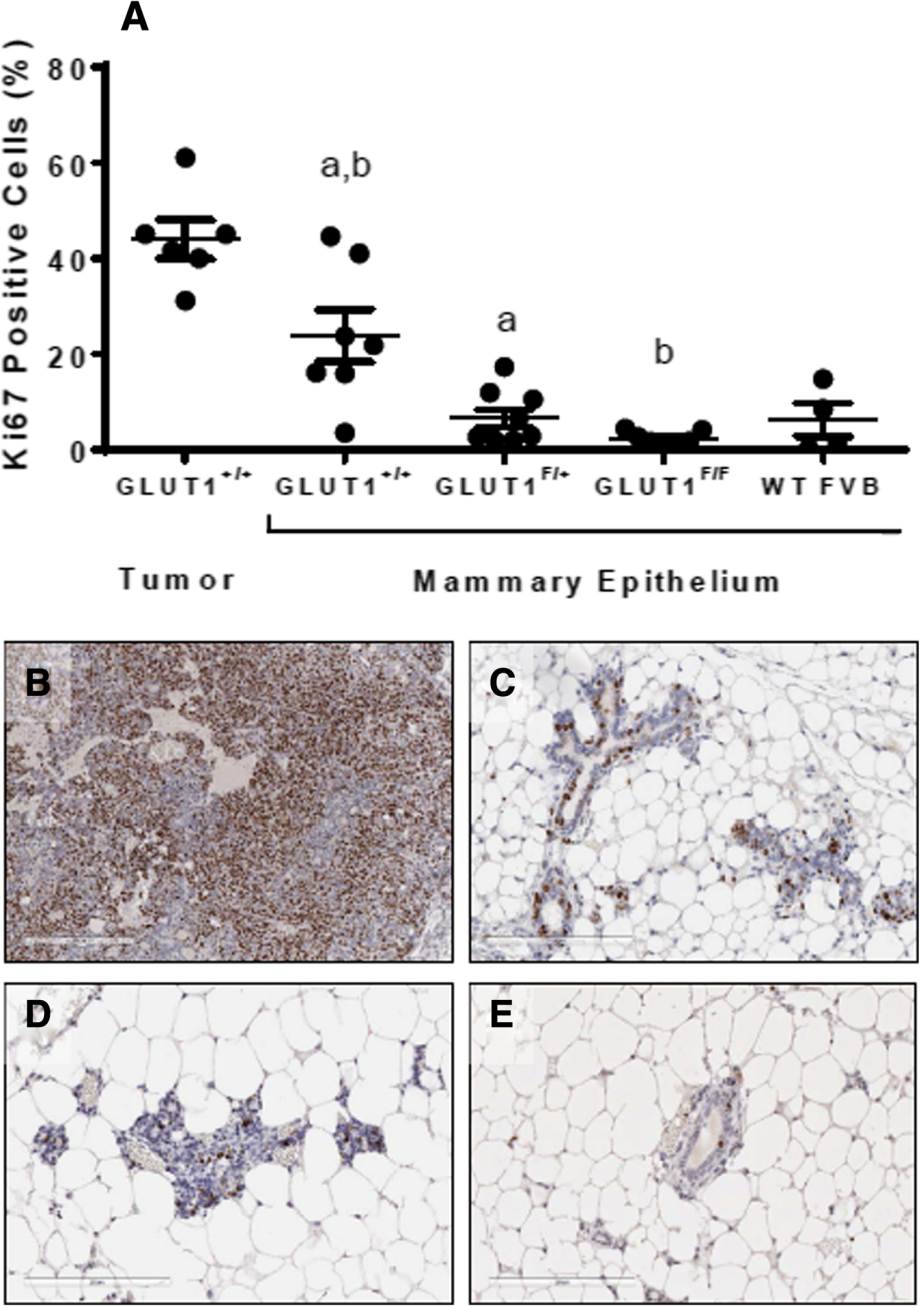
Reduced proliferation in mammary epithelium of mice lacking GLUT1. **a** Quantification of percent Ki67-positive epithelial cells in tumor tissue and in non-tumor-bearing mammary glands. Ki67 levels were significantly lower in NIC-GLUT1^F/+^ and NIC-GLUT1^F/F^ compared to NIC-GLUT1^+/+^ mammary epithelium. Groups with the same letter (*a, b*) are significantly different from each other, *p* < 0.05 by Mann-Whitney test. Ki67 levels in tumors and in mammary epithelium from wild-type (*WT*) FVB are shown for reference. Representative images of Ki67-stained tissue from a NIC-Glut1^+/+^ tumor (**b**) and non-tumor-bearing gland (**c**), and non-tumor-bearing glands from NIC-Glut1^F/+^ (**d**) and NIC-Glut1^F/F^ (**e**) mice. *Scale bars* for images are 200 μm

We evaluated Ki67 staining in tumors from NIC-GLUT1^+/+^ mice, and in mammary glands from NIC-GLUT1^+/+^, NIC-GLUT1^F/+^, and NIC-GLUT1^F/F^ mice to determine whether the differences in mammary gland morphology observed between genotypes were due to changes in proliferation. Tumors had the highest levels of Ki67-positive cells (~44%) when compared to the normal mammary epithelium from all mice (Fig. 3a and b). The normal mammary epithelium from NIC-GLUT1^F/+^ and NIC-GLUT1^F/F^ mice had lower levels of Ki67-positive cells compared to NIC-GLUT1^+/+^ mice (~6.5% and 2.3% versus 24%, respectively; Fig. 3a, c–e). Ki67 levels in wild-type, non-transgenic FVB control mice were approximately 6.2% (Fig. 3a), similar to NIC-GLUT1^F/+^ mice, indicating that loss of both *Slc2a1* alleles reduces normal mammary epithelial proliferation below that of wild-type mice. Overall, levels of the apoptotic marker, cleaved Caspase-3, were very low (<0.5% of mammary epithelial cell population) and were not different between groups (data not shown). These data suggest that a reduction in cell proliferation within the mammary epithelial compartment likely contributes to the observed differences in mammary gland structures with reduced or absent GLUT1.

### GLUT1 levels increase in early cancer cells and decrease as mammary tumors progress

**Fig. 4:**
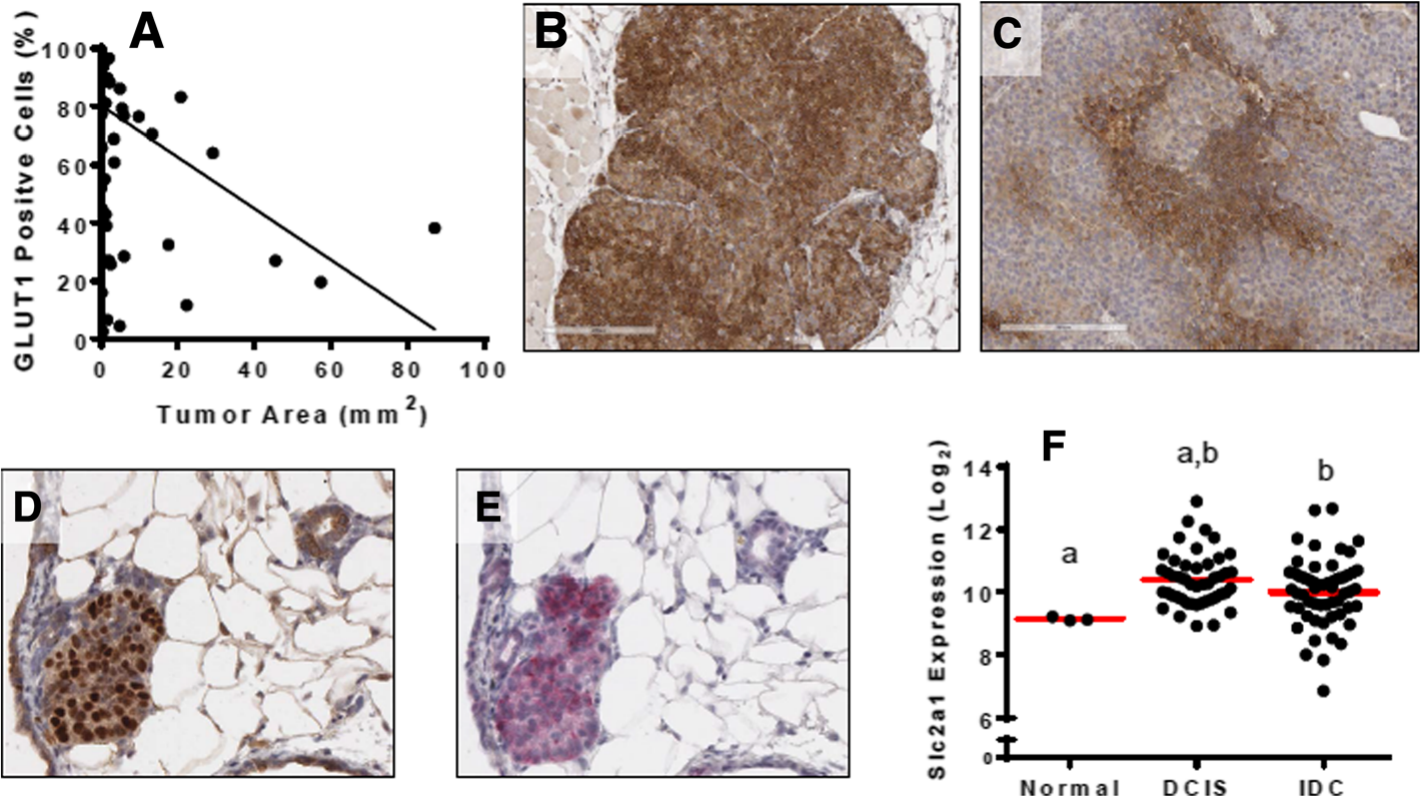
GLUT1 expression decreases with increasing tumor size. **a** GLUT1 protein levels are inversely correlated with tumor size. Spearman correlation *r* = –0.67; *p* < 0.0001; *N* = 81 tumors. Representative images of GLUT1 staining in small (**b**) and large (**c**) mammary tumors from NIC-GLUT1^+/+^ mice. *Scale bars* in images are 200 μm**.** Immunohistochemistry of Cre (**d**) and GLUT1 (**e**) expression in a small, non-palpable lesion in serial sections from a NIC-GLUT1^+/+^ mouse. **f** Analysis of *SLC2A1* expression in normal breast tissue (*Normal*), ductal carcinoma in situ (*DCIS*), and invasive ductal carcinoma (*IDC*) reported in GSE59246. Groups with the same letter (*a, b*) are significantly different from each other, *p* < 0.05 by unpaired *t* tests

Our previous study showed that shRNA knockdown or Cre-mediated excision of *Slc2a1* from cells that had previously grown as tumors in vivo only resulted in a partial inhibition of tumor growth [31]. This is in contrast to the near absolute requirement for *Slc2a1* in mammary tumorigenesis that we observed in this study (Fig. 1a). To determine whether GLUT1 changed during tumor growth, we examined GLUT1 protein levels in tumors of various sizes from NIC-GLUT1^+/+^ mice. Immunohistochemical analysis of GLUT1 levels in relation to tumor area showed a significant inverse correlation (*r* = –0.67; *p* < 0.0001) between tumor size and the percent of GLUT1-positive cells (Fig. 4a). Small tumors (<2 mm^2^) had more GLUT1-positive cells than larger tumors (Fig. 4b and c), suggesting that glucose uptake is required early during tumor formation, but that the tumor may become less dependent on GLUT1, and potentially on glucose, as it grows. Consistent with this, cells from small (non-palpable) masses with strong nuclear Cre expression showed elevation of GLUT1 (Fig. 4d and e). The observation that GLUT1 is elevated even in very small, non-palpable tumors indicates that GLUT1 upregulation may occur in response to oncogene activation and not necessarily in response to tumor hypoxia.

To extend these observations to human breast tumors, we evaluated *SLC2A1* expression in the normal breast, DCIS, and IDC samples from a publicly available gene expression dataset. *SLC2A1* expression was elevated in DCIS compared to normal breast samples, although the latter group contained only three samples. In contrast, *SLC2A1* expression was modestly but significantly reduced in IDC compared to DCIS tissues. This is consistent with an early requirement for GLUT1 that may decrease as breast tumors progress (Fig. 4f).

### Loss of GLUT1 influences mammary epithelial cell composition

**Fig. 5:**
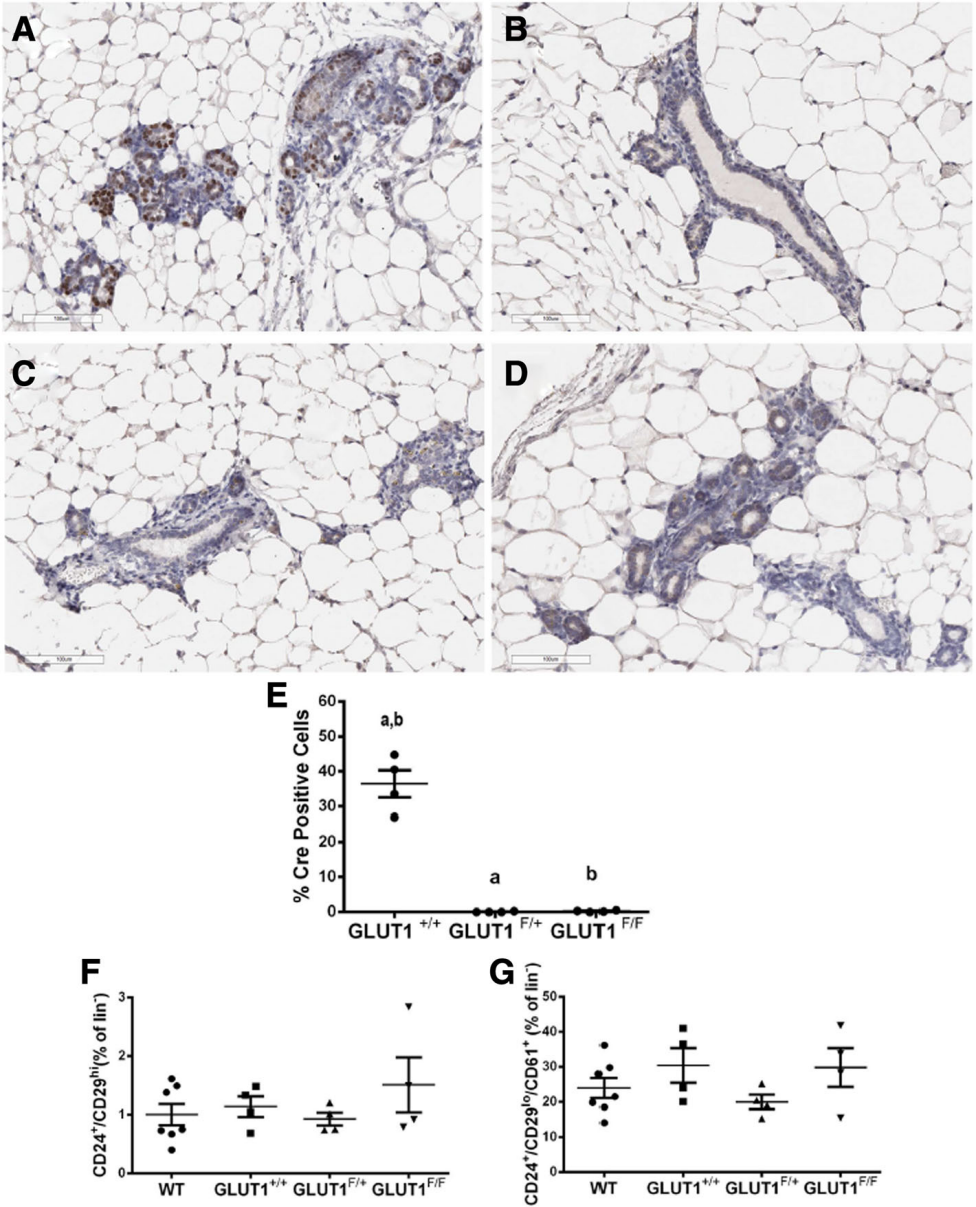
Nuclear Cre recombinase is not expressed in normal mammary epithelium of mice lacking GLUT1. Representative images of Cre staining from NIC-GLUT1^+/+^ (**a**), NIC-GLUT1^F/+^ (**b**), NIC-GLUT1^F/F^ (**c**), and wild-type FVB (**d**) mice. Inset in **d** is a wild-type FVB gland no-primary control. *Scale bars* for images are 100 μm. **e** Quantification of percent cells positive for nuclear Cre recombinase (*Cre*) in non-tumor-bearing mammary glands. Groups with the same letter (*a, b*) are significantly different from each other, *p* < 0.05 by Mann-Whitney tests. *N* = 4 mice per group. FACS analysis of mammary cell populations Lin-CD24^+^CD29^hi^ (**f**) and Lin-CD24^+^CD29^lo^CD61^+^ (**g**) from wild-type (*WT*) FVB (*N* = 7), NIC-GLUT1^+/+^ (*N* = 4), NIC-GLUT1^F/+^ (*N* = 4), and NIC-GLUT1^F/F^ (*N* = 4) mice

The dramatic reduction in mammary tumors seen with loss of GLUT1 suggested that the population of cells in which the NIC transgene is expressed may be eliminated from the mammary gland as the epithelium expands. We used immunohistochemical analysis to determine whether mammary glands from NIC-GLUT1^F/+^ and NIC-GLUT1^F/F^ mice expressed similar levels of Cre and GLUT1 as those from NIC-GLUT1^+/+^ mice. To our surprise, mammary glands from NIC-GLUT1^F/+^ and NIC-GLUT1^F/F^ mice had almost no cells with strong nuclear Cre expression (Fig. 5a). In NIC-GLUT1^+/+^ mammary glands, we saw strong nuclear Cre staining (Fig. 5a). In mammary glands from NIC-GLUT1^F/+^ and NIC-GLUT1^F/F^ mice, we detected almost no cells with Cre recombinase localized to the nucleus (Fig. 5b, c, and e). As a control, we performed immunohistochemical analysis of Cre in wild-type FVB mammary glands (Fig. 5d) and in mammary glands from NIC-negative, GLUT1 transgenic littermates (GLUT1^F/+^ and GLUT1^F/F^; data not shown) and observed no nuclear Cre staining in any of these groups. These data suggest that cells expressing strong nuclear Cre (i.e., Cre capable of excising the *Slc2a1* alleles) may be eliminated from the mammary epithelium, potentially once the Neu oncogene is induced, and GLUT1 is excised.

The hierarchy of mammary stem cells has been well characterized (reviewed in [36]). Cells expressing CD24, with low levels of CD29 (CD24^+^CD29^lo^) are thought to comprise the mature luminal cell type, while the CD24^+^CD29^hi^ population contains mammary stem cells. The surface marker CD61 (β1-integrin) is expressed in ductal or alveolar progenitor cells, and is lost as these cells mature [36]. We used flow-activated cell sorting (FACS) analysis to identify changes in mammary epithelial cell populations in NIC-GLUT1 transgenic mice and in wild-type FVB mice, using CD24, CD29, and CD61. Overall, we did not identify significant differences resulting from GLUT1 loss in CD24^+^/CD29^hi^ cells (Fig. 5f), nor did we detect significant differences in the luminal progenitor CD24^+^/CD29^lo^/CD61^+^ population (Fig. 5g). The MMTV promoter is reported to be expressed in luminal progenitor cells [37]; however, we did not see a depletion of this cell type consistent with it being eliminated from the gland.

### GLUT1 inhibition or glucose restriction blocks ErbB2-induced proliferation in vitro

**Fig. 6:**
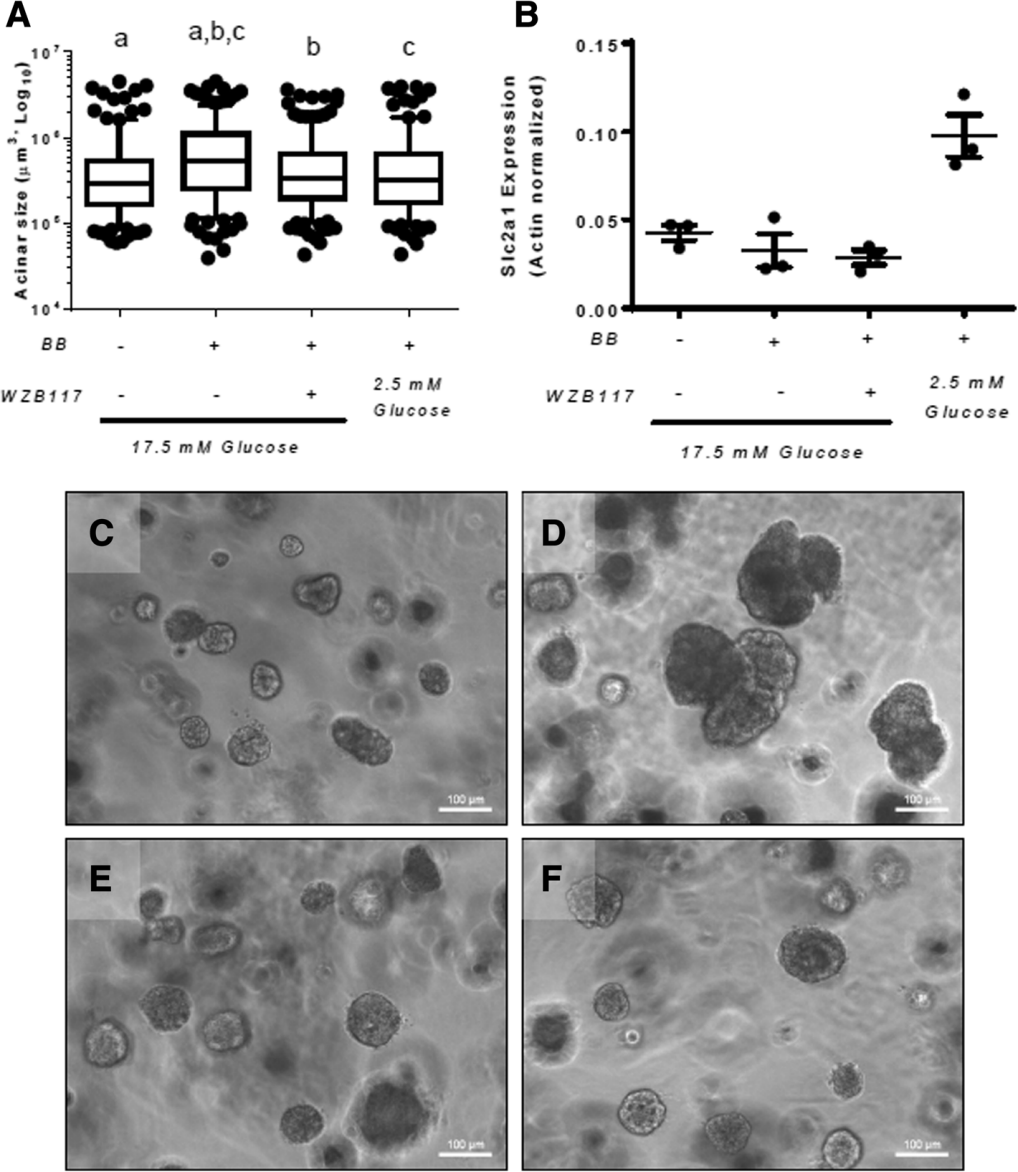
GLUT1 inhibition or glucose restriction prevents ERBB2-induced acinar growth. **a** Volumes (μm) of acinar structures grown in 17.5 mM or 2.5 mM glucose, with and without WZB117. Groups with the same letter (*a, b, c*) are significantly different from each other, *p* < 0.05 by Mann-Whitney tests. **b** Quantitative PCR analysis of *SLC2A1* in 3D acinar cultures under various treatments. Gene expression was normalized to *ACTB* (actin). Representative images, taken 13 days after seeding, of acinar structures grown in 17.5 mM glucose without BB homodimerizer to activate ERBB2 (**c**), with BB homodimerizer, added at day 6 of culture, in 17.5 mM glucose (**d**), with BB homodimerizer and 15 μM WZB117 added at day 6 of culture, in 17.5 mM media (**e**), or with BB homodimerizer added and switched to 2.5 mM glucose at day 6 of culture (**f**). *Scale bars* in images are 100 μm

**Fig. 7:**
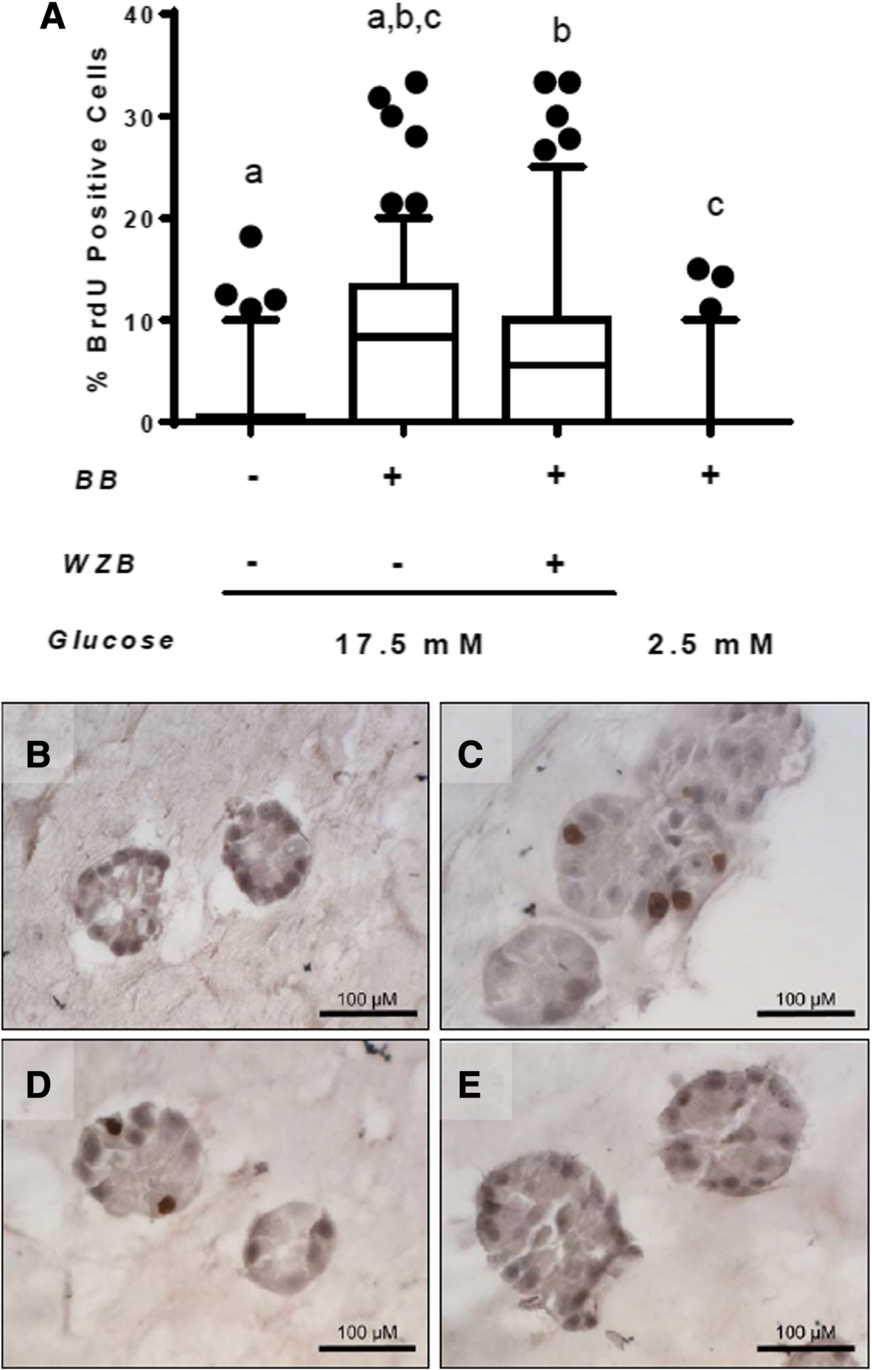
GLUT1 inhibition or glucose restriction reduces cell proliferation in 3D acinar structures. **a** Quantification of percent BrdU-positive cells in 3D acinar structures grown in 17.5 mM or 2.5 mM glucose, with and without WZB117. Groups with the same letter (*a, b, c*) are significantly different from each other, *p* < 0.05 by Mann-Whitney tests. Representative images, taken 8 days after seeding, of acinar structures grown in 17.5 mM glucose without BB homodimerizer to activate ERBB2 (**b**), with BB homodimerizer, added at day 6 of culture, in 17.5 mM glucose (**c**), with BB homodimerizer and 15 μM WZB117 added at day 6 of culture, in 17.5 mM media (**d**), or with BB homodimerizer added and switched to 2.5 mM glucose at day 6 of culture (**e**). *Scale bars* in images are 100 μm

To provide an in vitro correlate for the initial stages of tumorigenesis, we used the MCF10A-ERBB2 human breast epithelial cell line, which other investigators have used as a model to study the early events leading to transformation. In these cells, ERBB2 can be activated by addition of a chemical dimerizer to the culture media, leading to cellular transformation [34, 38]. When grown in three-dimensional (3D) cultures in the absence of the dimerizer, MCF10A-ERBB2 cells form hollow acinar structures that display apical/basal polarity [38, 39]. After ERBB2 dimerization is induced, the cells begin to proliferate and form multi-lobular structures that are not hollow and invade the surrounding matrix [34, 38, 39]. One hallmark of ERBB2 activation in this model is the formation of large acinar structures; thus, we determined the effect of GLUT1 inhibition, or glucose restriction, on 3D acinar growth in response to ERBB2 dimerization. The standard conditions for MCF10A cell growth use media containing 17.5 mM glucose [34, 38]. Activation of ERBB2 under these conditions significantly increased acinar size (Fig. 6a, c, and d; compare structures in 17.5 mM glucose treated with BB to untreated). To determine the effects of glucose restriction upon growth of MCF10-ERBB2 cells in 3D culture, cells were either treated with the GLUT1 inhibitor WZB117 [40], or the media was replaced with that containing 2.5 mM glucose at the time the “BB” dimer was added to the cultures. We did not observe an effect of BB dimer with or without WZB117 on expression of *SLC2A1*; however, glucose restriction (2.5 mM) increased *SLC2A1* expression in the presence of BB dimer (Fig. 6b), although these differences did not reach significance. Both GLUT1 inhibition and glucose restriction prevented the formation of large acinar structures in response to ERBB2 activation (Fig. 6a, e, and f). Analysis of BrdU incorporation revealed that activation of ERBB2 significantly increased cell proliferation in media containing 17.5 mM glucose (Fig. 7a–c). WZB117 addition modestly but significantly decreased cell proliferation in the presence of high glucose (Fig. 7a and d). The most dramatic reduction in cell proliferation was seen with glucose restriction to 2.5 mM (Fig. 7a and e). These data indicate that inhibition of GLUT1 or glucose restriction inhibits cell proliferation and de-regulates growth stimulated by active ERBB2.

## Discussion

In this study we have demonstrated that GLUT1 is required for tumor formation in a mouse model of Neu-induced mammary tumorigenesis. While it has been long appreciated that GLUT1 is overexpressed in mammary tumors, it is unclear when expression increases relative to normal breast. It has been suggested that GLUT1 levels increase with tumor size and reflect tumor hypoxia [41]. In addition, activated oncogenes such as Src, ERBB2, Akt, Ras, and Myc can drive expression of GLUT1 even under normoxic conditions. In our examination of the early preneoplastic lesions from NIC-GLUT1^+/+^ mice, GLUT1 levels were high in almost all cells and these cells also expressed Cre recombinase, which we used as a surrogate for expression of the Neu transgene (Fig. 4). As tumor size increased, GLUT1 levels decreased, with approximately 30% of tumor cells expressing high amounts of GLUT1 based on immunohistochemistry. Changes in *SLC2A1* expression from human tissues are similar; DCIS expressed higher *SLC2A1* than the normal breast, but levels were lower in IDC lesions (Fig. 4). These data are consistent with our previous work, which demonstrated that *Slc2a1* knockdown or Cre-mediated excision from mammary tumor cell lines that had grown as tumors in vivo only reduced tumor growth by 50% [31]. Conversely, when *Slc2a1* was excised from PyMT-transformed mammary epithelial cells prior to orthotopic xenograft injection, no tumors formed [31]. The decreased expression of GLUT1 in larger tumors suggests that the requirement for GLUT1 and glucose may change as the tumor progresses; however, the continued use of PET scanning demonstrates that glucose is still utilized by advanced tumors.

In this study, we found that loss of a single copy of *Slc2a1* is sufficient to prevent tumorigenesis (Fig. 1). A growing number of studies have been reported using the NIC mice and none have observed haplo-insufficiency for genes required for Neu-induced mammary tumorigenesis. Genes examined for their role in ERBB2-mediated mammary tumorigenesis include ShcA [32], β-catenin [42], Bcl3 [43], hexokinase II [44], DOCK1 [45], LKB1 [46], 14-3-3σ [47], STAT1 [48], and specific isoforms of phosphatidylinositol 3’-kinase [49]. The haplo-insufficiency observed in our study indicates a strong requirement for GLUT1 and glucose in mammary tumorigenesis. This may not be surprising given the nature of GLUT1 deficiency syndrome (GLUT1-DS), an autosomal dominant haplo-insufficiency disorder that results from decreased glucose transport to the brain [50]. Patients with GLUT1-DS display motor and developmental delays, infantile onset of drug-resistant seizures, and deceleration of head growth resulting in acquired microcephaly, ataxia, and dystonia, [50]. Patients have decreased glucose levels in cerebrospinal fluid with normal blood glucose levels. Our studies would suggest that GLUT1-DS patients may be naturally resistant to many types of cancer.

In our model, one NIC-GLUT1^F/+^ and one NIC-GLUT1^F/F^ mouse developed mammary tumors. The tumors from the NIC-GLUT1^F/+^ mouse expressed Cre and GLUT1 (Fig. 2), suggesting that, in this mouse, *Slc2a1* haplo-insufficiency did not occur, and that there may have been early changes in tumor metabolism that overcame the loss of one *Slc2a1* allele. Interestingly, all of the tumors from the NIC-GLUT1^F/F^ mouse expressed nuclear Cre; however, none expressed GLUT1. The tumors in this mouse may harbor mutations in metabolic genes that allow progression in the absence of GLUT1. Our future studies will address the possibility of a compensatory increase in other glucose transporters as well as potential novel mutations in metabolic genes that allow tumors to emerge without upregulating GLUT1.

Strong nuclear Cre expression was not observed in non-tumor-bearing mammary glands from NIC-GLUT1^F/+^ or NIC-GLUT1^F/F^ mice (Fig. 5). This suggests that, once the HER2/ERBB2/Neu oncogene is expressed, the requirement for GLUT1 may not be met even with the loss of one allele. These cells may die, or may undergo senescence and be overgrown by other cell types in the mammary epithelium. We attempted to identify differences in the luminal cell populations within these mammary glands using FACS analysis; however, we were unable to demonstrate a reduction in the luminal progenitor cell population. It is possible that the targets of transformation in MMTV-NIC mice are indicated by alternative cell markers than those we evaluated.

To extend our in vivo observations, we examined the effects of GLUT1 inhibition or glucose restriction on acinar size in ERBB2-overexpressing MCF10A cells (Fig. 6). The MCF10A-ERBB2 cell line is well characterized and forms structures reminiscent of normal mammary epithelial structures when grown in a 3D matrix. After ERBB2 dimerization is induced, these structures proliferate and invade, much like breast cancer lesions in patients [39]. Inhibition of GLUT1 using a small molecule inhibitor or glucose restriction in the culture media suppressed ERBB2-induced transformation of MCF10A cells, measured by increased acinar size. This suggests that it is a decrease in glucose entering tumor cells, rather than the presence of GLUT1 itself, that suppresses tumorigenesis. These results are consistent with those reported in the HMT-3522 breast cell lines. This series includes non-malignant (S1) and malignant (T4-2) breast cells derived from a single reduction mammoplasty. In this model, GLUT1 levels were not different between S1 and T4-2 cells, but GLUT3 increased dramatically in malignant compared to benign cells [51]. In addition, GLUT3 inhibition as well as glucose restriction prevented the formation of proliferative, non-polarized acinar structures [51]. Together, our data from MCF10A-ERBB2 cells and the previously published study performed on HMT-3522 cells implicate a role for glucose in the process of breast epithelial transformation and underscore the importance of elucidating how cancer cell metabolism changes during tumor progression to develop optimal therapies.

The utility of targeting GLUT1 in the treatment of cancer has been explored by other investigators. Rastogi et al. demonstrated that treatment of two established breast cancer cell lines, MCF7 and T47D, with anti-GLUT1 antibody in vitro caused up to a 75% reduction in proliferation [52]. Antisense RNA targeting GLUT5 has proved effective against two breast cancer cells lines expressing this transporter [53]. Small molecule inhibitors specific for GLUT1, including WZB117 which we have utilized in our studies, have been identified [54], as have inhibitors for other transporters such as GLUT5 [55]. These molecules offer the opportunity to selectively target specific transporters as part of single or combination therapies.

Clearly our studies have utilized a single mouse model, although it does express an oncogene that is relevant to human breast cancer. We will extend these studies to other mouse models of mammary tumorigenesis dependent upon other oncogenes or tumor suppressor genes. In addition, we are interested in determining whether mouse models of other types of cancer (e.g., lung, head, and neck) are also dependent upon GLUT1.

There is an abundance of literature on cancer cell metabolism reporting that tumor cells are similarly addicted to glutamine, and that glutamine can replace glucose to meet the bioenergetic and biosynthetic needs during cell growth [56–62]. Many of these studies were conducted using established cell lines that have been in culture for years, if not decades, and heavy-atom nutrient tracers to follow glutamine utilization. While these studies are informative, it is possible that primary tumor cells in vivo have different metabolic requirements than established cell lines. Indeed, it was recently reported that Ras-driven non-small-cell lung cancer displays marked differences in metabolism when grown as orthotopic xenografts as compared to two-dimensional culture [63]. Specifically, cells grown in vitro utilized glucose and glutamine for carbon building blocks, while cells grown in vivo appeared to rely almost exclusively on glucose [63]. Moreover, none of the studies on glutamine address whether it can meet the metabolic needs of the cells during the initial stages of tumorigenesis. Our study suggests that, in the absence of GLUT1 and glucose uptake, early transformed cells may not be able to use glutamine to meet their proliferative demands. Cells within a newly formed tumor may be metabolically inflexible, and the ability to use other carbon sources may change as the tumor progresses in the host environment. This would suggest that a preventive strategy could be taken focused entirely on modulating glucose uptake and metabolism at the earliest stages of tumorigenesis.

In conclusion, our data clearly indicate an important and unique role for the GLUT1 transporter, and by extension glucose, in the earliest stages of Neu-induced mammary tumorigenesis in vivo, which cannot be replaced by other transporters or carbon sources. Despite our increased understanding of various carbon sources and biochemical pathways that can contribute to the altered metabolism observed in tumor cells, it appears that glucose metabolism remains central to the beginnings of tumor development. These other pathways and carbon sources may become increasingly important as tumors evolve; however, glucose appears to be uniquely important in the initial stages of tumorigenesis. Furthermore, this unique role for glucose suggests the potential for chemoprevention strategies that restrict available glucose or block tumor glucose uptake.

## Conclusions

Altered tumor cell metabolism has emerged as a hallmark of cancer [59]. Many studies have identified novel metabolic networks and substrates that support the proliferation of established cancer cells in culture. Our data show, in a conditional mouse model, that the glucose transporter GLUT1 is required for the formation of mammary tumors and for robust ERBB2-induced proliferation in a 3D model of malignant transformation. This study indicates that early tumor cells have not acquired the ability to utilize alternative substrates for growth, and highlights the potential to focus on glucose restriction as a preventive strategy for breast cancer.

## Abbreviations

DCIS: Ductal carcinoma in situ
FACS: Flow-activated cell sorting
FDG: ^18^F-fluoro-2-deoxyglucose
HER2/ErbB2/Neu: Human epidermal growth factor receptor family member 2
IDC: Invasive ductal carcinoma
IRES: Internal ribosome entry site
MMTV: Mouse mammary tumor virus
NADPH: Nicotinamide adenine dinucleotide phosphate
NIC: Neu-IRES-Cre
PET: Positron emission tomography
PyMT: Polyomavirus middle-T antigen
STR: Short tandem repeat
